# Systematic review of the association between adverse life events and the onset and relapse of postpartum psychosis

**DOI:** 10.3389/fpsyt.2023.1154557

**Published:** 2023-04-17

**Authors:** Thomas J. Reilly, Emma Roberts, Vanessa Charlotte Sagnay De La Bastida, Philip McGuire, Paola Dazzan, Alexis E. Cullen

**Affiliations:** ^1^Department of Psychiatry, Medical Sciences Division, University of Oxford, Oxford, United Kingdom; ^2^Department of Psychosis Studies, Institute of Psychiatry, Psychology & Neuroscience, King’s College London, London, United Kingdom; ^3^NIHR Oxford Health Biomedical Research Centre, Oxford, United Kingdom; ^4^Department of Psychological Medicine, Institute of Psychiatry, Psychology & Neuroscience, King’s College London, London, United Kingdom; ^5^Division of Insurance Medicine, Department of Clinical Neuroscience, Karolinska Institutet, Stockholm, Sweden

**Keywords:** psychotic disorders, schizophrenia, postpartum, perinatal, childbirth, adverse childhood experiences, stress, trauma

## Abstract

Postpartum psychosis is defined as a psychotic episode occurring within 4 to 6  weeks of childbirth. While there is robust evidence that adverse life events are associated with the onset and relapse of psychosis outside the postpartum period, the extent to which these contribute to postpartum psychosis is less clear. This systematic review examined whether adverse life events are associated with an increased likelihood of developing postpartum psychosis or subsequent relapse in women diagnosed with postpartum psychosis. The following databases were searched from inception to June 2021: MEDLINE, EMBASE, PsycInfo. Study level data were extracted including setting, number of participants, type of adverse event, and differences between groups. A modified version of the Newcastle-Ottawa Quality Assessments Scale was used to assess risk of bias. In total, 1933 records were identified, of which 17 met the inclusion criteria, comprising nine case–control studies and eight cohort studies. Most studies (16/17) examined the association between adverse life events and the onset of postpartum psychosis, with only in which the outcome was relapse of psychosis. Overall, there were 63 different measures of adversity examined (most of which were examined in a single study only) and 87 associations between these measures and postpartum psychosis tested across the studies. In terms of statistically significant associations with onset/relapse of postpartum psychosis, 15 (17%) were positive (i.e., the adverse event increased the risk of onset/relapse), 4 (5%) were negative, and 68 (78%) were not statistically significant. Our review highlights the diversity of risk factors examined in this field, with few attempts at replication, hence limiting the ability to conclude that any single risk factor is robustly associated with the onset of postpartum psychosis. Further large-scale studies, that attempt to replicate earlier studies, are urgently needed to determine whether adverse life events play a role in the onset and exacerbation of postpartum psychosis.

**Systematic review registration:**

[https://www.crd.york.ac.uk/prospero/display_record.php?RecordID=260592], identifier [CRD42021260592].

## 1. Introduction

Postpartum psychosis is a psychiatric emergency (1) that occurs in 0.9 to 2.6 per 1,000 births (2). Variation in the definition of postpartum psychosis across studies has prevented a global estimate of prevalence and meaningful comparison across countries (2). The risk of illness onset is higher among women with an existing diagnosis of bipolar disorder, those who have experienced postpartum psychosis following a previous pregnancy, and in primiparous women (3). Postpartum psychosis typically presents within 10–19 days of birth, with the most common presentation being a combination of affective and psychotic symptoms (4). The nosology of postpartum psychosis is not clear but the disorder is generally considered to fall within the bipolar spectrum (5), with overlapping symptomatology (6). Indeed, bipolar disorder has the highest risk of relapse within the postpartum period (7).

While postpartum psychosis is relatively rare, it is associated with severe outcomes: women with postpartum psychosis are at increased risk of suicide (8), a leading cause of maternal death in the year following delivery (9). With respect to illness course, most women experiencing postpartum psychosis do not have a previous history of mental illness (10), yet more than half later experience relapses that are not associated with childbirth (11). Compared to psychotic episodes that occur during other periods, postpartum psychosis has a clear triggering event, childbirth. However, the reasons why a small subgroup of women are particularly vulnerable to developing an episode of psychosis following birth are poorly understood.

Outside the postpartum period, psychotic disorders are thought to arise through a complex interaction of genetic (12), developmental (13), and environmental risk factors (14). Adverse life events form a category of environmental risk factors, broadly defined as exposure to difficult or unpleasant situations or experiences (15), that can occur during childhood or adulthood. Childhood adverse life events most frequently studied in relation to psychosis include household poverty, parental separation, death of a parent, neglect, abuse, and bullying (15). In adulthood, adverse events may include relationship breakdown, employment difficulties, death of a loved one, and housing problems (16). Some adverse events are specific to the postpartum period, such as complications of pregnancy, complications of childbirth, and complications in the neonate. Although these events are not typically included as ‘adverse events’ in the body of psychosis research, there is evidence that they are psychologically distressing for the mother (17) and might therefore be an important risk factor for postpartum psychosis.

While there is consistent evidence indicating that adverse life events are associated with psychosis onset in both males and females (16) and with illness relapse (18), previous narrative reviews (4, 19, 20), have found limited evidence to suggest that adverse life events or social stress are significant risk factors for postpartum psychosis. In contrast, other postpartum disorders such as depression are robustly associated with adverse life events (21) and perinatal complications (22). Given the contribution of adverse life events to development of (non-postpartum) psychosis and postpartum depression, a systematic appraisal of their role in the onset of postpartum psychosis in warranted. We are aware of one systematic review that examined peripartum complications in relation to first onset postpartum psychosis (23): the authors identified various risk factors from single studies but included case reports and case series (which could introduce bias) and did not utilize a risk of bias tool to assess this in a structured way.

To address the gap in current knowledge, we aimed to systematically review and appraise the quality of evidence for the association of adverse life events with (i) new onset of postpartum psychosis, and (ii) subsequent relapse of psychosis (irrespective of whether relapse occurred following childbirth) in women previously diagnosed with postpartum psychosis. We defined adverse life events as any event that could be considered as a stressor, ranging from individual events occurring in childhood or adulthood, those assessed using life event scales/checklists, and perinatal complications.

## 2. Materials and methods

PRISMA guidelines for systematic reviews were followed (24), see Supplementary material for checklist, and pre-registered with PROSPERO (CRD42021260592).

### 2.1. Search strategy

The following databases were searched from inception until June 2021: MEDLINE, EMBASE, PsychInfo. References from a recent review (23) examining associations between obstetric complications and postpartum psychosis were searched. Titles, abstracts, and keywords were searched using the combinations of terms shown below, with the full search strategy outlined in the Supplementary Materials:

- Puerper* OR Postpart* OR Post-part* OR After-pregnan* OR Childbirth* OR Child-birth* OR New-mother* OR Labor OR Labour OR Cesarean OR Delivery OR Deliveries OR Postnatal* OR Post-natal* OR Parturition
- Psychosis* OR Psychotic* OR Psychoses* OR Bipolar OR Mania*
- Life event OR Stress* OR Trauma* OR Advers* OR Abus*

Two authors (ER, VCSB) screened study titles and abstracts of all articles identified in the search and retrieved potentially relevant full-text articles for detailed examination. Full-text publications were then independently reviewed against inclusion criteria by a minimum or two authors (ER, VCSB, TR). Disagreements were resolved by the senior author (AEC).

### 2.2. Inclusion criteria

Studies were included if they measured the association between adverse life events and first onset of postpartum psychosis or subsequent relapse (either within or outside the postpartum period) among those who had a history of postpartum psychosis. Both prospective and retrospective studies were included, with no restrictions based on language. Studies examining risk factors for first onset postpartum psychosis were excluded if they did not include a control group of healthy women, or if they examined a range of postpartum psychiatric disorders without a clearly defined subgroup with postpartum psychosis. Studies examining factors associated with psychosis relapse among women with postpartum psychosis were excluded if they did not include a non-relapsed comparison group. Case studies, reviews, and publications that had not undergone peer reviewed (dissertations and conference abstracts) were not eligible for inclusion.

First-onset postpartum psychosis was defined as the first psychotic episode occurring during the postpartum period. Given variability in the length of this period across studies, and to maximize the number of studies eligible for inclusion, we considered any definition of ‘postpartum’ used by the original study authors. This could be longer than the definitions applied in the most recent versions of the Diagnostic and Statistical Manual [DSM-5, (25)] or the International Classification of Diseases [ICD-11, (26)] which require onset within 4 and 6 weeks after birth, respectively. Relapse in patients with a prior diagnosis of postpartum psychosis was defined as any subsequent psychotic episode (either within, or outside of, any subsequent postpartum period), as indicated by exacerbation of psychotic symptoms, clinical diagnosis of psychotic episode, readmission to hospital, or a combination of these.

There were no restrictions on the type of adverse life events; these encompassed abuse (physical, emotional, sexual or neglect), stressful life events, trauma, or other adverse experiences. In addition to conventionally defined adverse life events, any complication relating to pregnancy, childbirth, or the neonate was also considered an adverse life event as these complications are known to be psychologically distressing and are associated with postpartum depression.

### 2.3. Data extraction

Two authors (ER, TJR) extracted study level data independently, with any discrepancies discussed with the senior author (AEC). The following variables were extracted for each study: study setting, number of participants, mean age, length of postpartum period, type of adverse event measured, and study results. For studies using a scale or questionnaire to assess adverse events, the mean and standard deviation (SD) were extracted. For longitudinal studies examining associations between risk factors and outcome, the incidence rate ratio (IRR), relative risk (RR), odds ratio (OR) or hazard ratio (HR) were extracted. For those comparing the number of adverse events between groups, the absolute number, median and interquartile range (IQR) were extracted. For studies with overlapping samples (27, 28), the study with the most complete relevant dataset was included, with authors contacted if this was not clear.

The two pre-specified primary outcome measures were (i) the development of first-onset postpartum psychosis and (ii) relapse of psychosis among women with a history of postpartum psychosis. We classified adverse life events as: complications relating to childbirth, complications of pregnancy, complications in the neonate, individual adverse events experienced by the mother in both childhood and adulthood, and scales measuring life events.

### 2.4. Assessment of study quality

Modified versions of the Newcastle-Ottawa Quality Assessment Scale for case–control and cohort studies (29) were used to assess the risk of bias. These scales assess study quality in the domains of selection of participants, comparability of groups, and measurement of outcome. Ratings were performed independently by a minimum of two authors (ER, TJR, AEC) with disagreement resolved by discussion. The maximum possible score was 12 for both case–control and cohort designs.

## 3. Results

**Figure 1:**
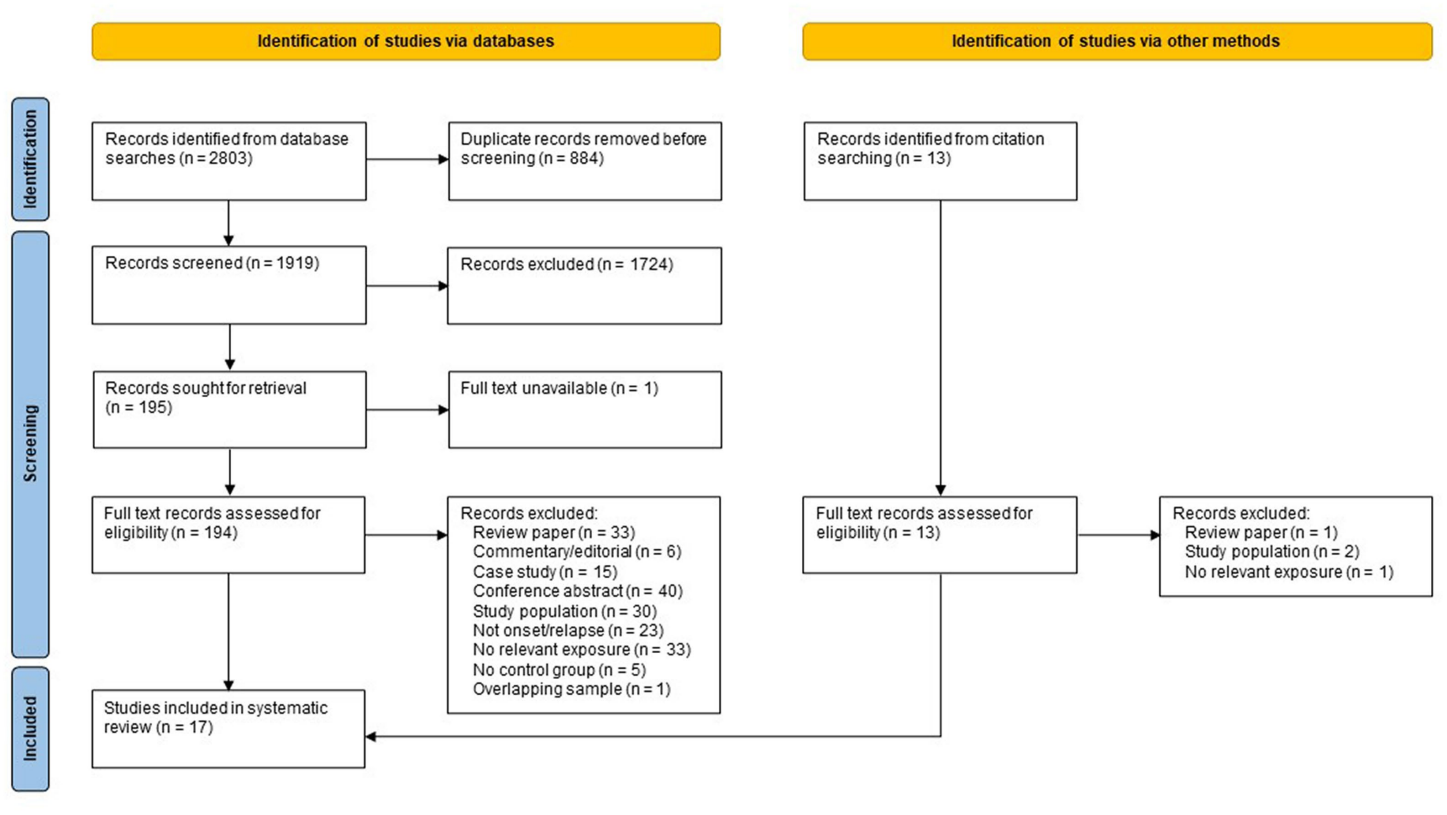
PRISMA flow diagram of study selection.

Searching of electronic databases and manual citation searching yielded 1,933 records after deduplication (see Figure 1), of which 17 met the inclusion criteria (27, 30–45), representing 18 independent study samples. Of the 17 included studies, eight were identified *via* database searching (27, 30, 32, 33, 35, 37, 44, 45), five were identified from a recent systematic review by Nguyen et al. (34, 38, 40, 42, 43) and four studies were found by citation searching of included references (31, 36, 39, 41). Five samples were from Denmark, four from the UK, three from Sweden, three from North America, one from South Africa, one from the Netherlands, and one from India. Nine studies used a case–control design and eight used a cohort design. The earliest study was published in 1960, with most published after 2000. All but one of these studies (16/17) examined the association between adverse life events and new onset of postpartum psychosis; only one study (41) examined the association between adverse life events and subsequent relapse among women previously diagnosed with postpartum psychosis.

Characteristics and findings of the nine case–control studies are summarized in Table 1. The total sample size of case–control studies was 1,603, with a mean study sample size of 178 (SD = 152). The total number of participants with postpartum psychosis was 821 (mean = 91; SD = 115). Six studies measured exposure to obstetric complications (obtained from medical records), and three used checklists to measure exposure to psychosocial stressors. All studies compared women diagnosed with postpartum psychosis (cases) to healthy postpartum women (controls), while Aas et al. (27) additionally included a group who were at risk of postpartum psychosis (due to history of bipolar disorder or schizoaffective disorder) but remained well in the postpartum period.

**Table 1:** Summary of case–control studies examining association with onset of postpartum psychosis.

Study	Number of participants	Percentage first episode	Postpartum period	Diagnosis	Country	Setting	Adverse event	Measures	Findings
Aas 2020 (27)	56 (14 postpartum psychosis, 16 at risk of postpartum psychosis who remained well, 26 healthy controls)	71	4 weeks	DSM-IV, SCID	UK	Perinatal services in two London hospital trusts	Recent stressful life events	List of Threatening Experiences questionnaire	Mean LTE score 3.5 (SD 3.4) in postpartum psychosis group, 2.2 (SD 3.2) in at risk group who did not develop postpartum psychosis, 0.7 (SD 1.7) in healthy controls. Significant difference between groups (F = 4.3, p = 0.019), post hoc tests showed that the postpartum psychosis group was significantly different from healthy controls (*p* = 0.007).
Allwood 2000 (30)	489 (381 postpartum psychosis, 98 healthy controls)	78	90 days	DSM-III-R	South Africa	Department of Psychiatry at Baragwanath Hospital, cohort was of Black African women	Psychosocial stressors	Severity of Psychosocial Stressors Scale rated by clinicians using medical records; clinical file review to determine mode of delivery, length of hospital stay, and complications	No significant differences in the Severity of Psychosocial Stressors Scale. Higher incidence of Cesarean sections in the healthy control group (may be an artifact of the follow-up, as control patients who had natural deliveries were less likely to return for assessment). The prospective postpartum psychosis group had longer (mean = 7.4 days) hospital stays than healthy controls (mean = 3.5 days, *p* < 0.001). A higher proportion of babies needed intensive care stay in the prospective postpartum psychosis group (40.7%) than the healthy control group (24.5%, *p* < 0.01).
Bergink 2013 (31)	119 (63 postpartum psychosis, 56 healthy controls)	100	4 weeks	DSM-IV-TR, SCID	Netherlands	Mother and baby unit, Department of Psychiatry of Erasmus University Medical Center, Rotterdam	Cesarean Section	Not reported	6/63 (9.5%) women in the postpartum psychosis group had Cesarean section, compared with 14/65 (25%) of healthy controls. This was statistically significant (Fisher exact test, *p* = 0.03). 6/63 (9.5%) and 7/56 (9.3%) of postpartum psychosis and controls had vacuum extraction, differences were not statistically significant (Fisher’s exact, *p* = 0.77).
Brockington 1990 (32)	113 (33 postpartum psychosis, 80 healthy controls)	100	1 month	Research diagnostic criteria	UK	Mother and baby unit at South Manchester University Hospital	Recent life events	Life Events and Difficulties Schedule	15% of the postpartum psychosis group experienced a ‘provoking agent’ (defined as a markedly or moderately threatening life event or difficulties that were rated as sufficiently severe) in the 38 weeks prior to interview compared with 36% of the community sample (Chi-squared test = 4.94, *p* = 0.026).
Dowlatshahi 1990 (33)	66 (33 postpartum psychosis, 33 healthy controls)	Not reported	1 month	ICD-9	UK	Two Mother and Baby Units in South London	Recent life events	Interview for Recent Life Events	No significant differences between groups in the overall number of recent life events in the postpartum psychosis group (mean = 2.67) compared to controls (mean = 3.36), test statistics and *p* values not reported. No differences when each type of event examined separately. Among those with postpartum psychosis, no differences between the 12 patients who also fulfilled depression criteria (mean = 2.17) and the 21 patients those who did not (mean = 2.95).
Paffenbarger 1961 (39)	378 (126 postpartum psychosis, 252 healthy controls)	100	6 months	Not reported	USA	Cincinnati, Ohio.	Obstetric complications	Hospital records	Significantly more women with postpartum psychosis experienced maternal respiratory illness than healthy women, 9% vs. 3% (Chi-squared test *p* < 0.05). Total dystocia was also significantly more common in women with postpartum psychosis than healthy women, 14% vs. 5% (Chi-squared test, *p* < 0.05) as was fetal/neonatal death, 7% vs. 3% (Chi-squared test, *p* = 0.02). No significant differences between postpartum psychosis and healthy women were found for pre-eclampsia, 9% vs. 3%; placenta previa, 1% vs. 1%; abruptio placentae, 1% vs. 1%; prolapsed cord, 2% vs. 0%; malpresentation, 10% vs. 5%; cephalopevlic disproportion, 2% vs. 0%; uterine dysfunction, 5% vs. 0%.
Sharma 2004 (40)	42 (21 postpartum psychosis, 21 healthy controls, note only 34 participants had duration of labour data)	Not reported	4 weeks	DSM-IV	Canada	Two hospitals in London, Ontario and a third in the town of Woodstock	Duration of labour and night-time delivery	Case note review	Mean duration of labour 11.15 h in the postpartum psychosis group and 7.34 h in the control group (one sided t test, *p* < 0.05). 71% of the postpartum psychosis group had night-time delivery compared to 41% of the control group (Chi-squared test, *p* < 0.05).
Upadhyaya 2014 (42)	200 (100 postpartum psychosis, 100 healthy controls)	100	42 days	DSM-IV-TR	India	Government tertiary care hospital in rural Uttarakhand,	Obstetric complications	Questionnaire (not well described)	No significant difference in Cesarean/instrumental delivery, 37% in postpartum psychosis, 18% in controls (Chi-squared test, *p* = 0.12). No significant difference in any maternal complication during pregnancy, 36% in postpartum psychosis, 24% in healthy controls (Chi-squared test, *p* = 0.06). Any maternal complication during the perinatal period was significantly more common in the postpartum psychosis group than in the healthy control group, 38% vs. 22% (Chi-squared test, *p* = 0.01). Significantly more complications in the new-born, 21% in postpartum psychosis, 8% in healthy controls (Chi-squared test, *p* = 0.009).
Videbech 1995 (43)	150 (50 postpartum psychosis, 100 healthy controls)	100	1 year	ICD-8 and DSM-IV	Denmark	The Danish Psychiatric Central Register and the Danish Medical Birth Register were linked to identify all women admitted for the first time to a psychiatric department in Arhus County with a psychotic episode	Obstetric complications	Hospital records	Preterm birth occurred in 27% of postpartum cases vs. 9% of controls; after adjusting for parity, this was statistically significant (OR = 3.6, p < 0.01). Mean birth weight significantly lower in women with postpartum psychosis compared to controls (3,204 g vs. 3,523 g, Chi-squared = 10.78, p < 0.05). No significant differences in post-term delivery or birth duration. Complications during pregnancy (such as bleeding or uterine contractions leading to sick leave) occurred with equal frequency in the cases and the controls. The birth complications and surgical interventions such as acute Cesarean section did not occur more frequently than expected among the probands.

DSM, Diagnostic and Statistical Manual; SCID, Structured Clinical Interview for DSM; LTE, List of Threatening Experiences; SD, Standard Deviation, International Classification of Disease.

Findings from the seven cohort studies examining onset of postpartum psychosis are summarized in Table 2. Five were population-based registry studies, based in either Denmark or Sweden. The mean sample size across all cohort studies was 1,007,586 (SD = 1,483,998) and the mean number of cases of postpartum psychosis 1,147 (SD = 1,712). There was potential overlap across the study samples, with Meltzer-Brody et al. (37), Meltzer-Brody et al. (36), and Warselius et al. (45) using national registry data from Denmark to measure the association of different exposures. While Nager et al. (38), Vikstrom et al. (44), and Warselius et al. (45) used overlapping samples of the Swedish population. However, as all studies with potentially overlapping samples measured different exposures, all were included in the review. Findings from the single study (41) examining subsequent relapse in women previously diagnosed with postpartum psychosis are summarized in Table 3.

**Table 2:** Summary of cohort studies examining onset of postpartum psychosis.

Study	Number of participants	Percentage first episode		Postpartum period	Diagnosis	Country	Setting	Adverse event	Measures	Findings
Kendell 1981 (34)	35,800 (71 postpartum psychosis)	Not reported		90 days	ICD-8	UK	All women who were admitted to a psychiatric hospital (Royal Edinburgh Hospital) within 90 days of childbirth. Data taken from all births in Edinburgh between 1971 and 1977.	Obstetric complications	Hospital records	21.6% of the postpartum psychosis group had Cesarean section, compared with 8.4% of the healthy controls, Chi-squared test, significant at the 1% level. Other variables that were not statistically significant - perinatal death 2.8% vs. 1.7%, forceps delivery 16.9% vs. 18.7%, antepartum hemorrhage 4.2% vs. 2.3%, anemia in pregnancy 1.4% vs. 3.2%, malposition *in utero* 2.8% vs. 0.9%, hydramnios 0% vs. 1.1%, renal/urinary infection 8.4% vs. 6.4%, pre-eclampsia 7.0% vs. 11.3%, post-partum hemorrhage 0% vs. 4.9%, abnormality of bony pelvis 0% vs. 1.2%, malpresentation of fetus 2.3% vs. 1.3%, prolonged labor 1.4% vs. 3.5%, laceration of perineum 2.8% vs. 3.5%, sepsis 2.8% vs. 3.4%, pyrexia of unknown origin 1.4% vs. 1.8%, puerperal anemia 2.8% vs. 1.8%.
Lewkowitz 2019 (35)	1,203,050 (4,746 postpartum psychosis)	100		1 year	ICD-9	USA	Florida State Inpatient Database and Emergency Department Database of the Agency for Healthcare Research and Quality’s Healthcare Cost and Utilization Project from 2005–2015, including all in-hospital deliveries from the third quarter of 2005 through December 31, 2014.	Stillbirth at >23 weeks’ gestation	Hospital records	0.9% of mothers who experienced stillbirth developed postpartum psychosis vs. 0.4% in the livebirth group. Stillbirth associated with postpartum psychosis in multivariable model [OR = 2.27 (95% CI: 1.79–2.88)] adjusted for maternal age, race/ethnicity, insurance type, income quartile by zip code, mode of delivery, severe intrapartum maternal morbidity, and medical comorbidities.
Meltzer-Brody 2017 (37)	392,458 (172 postpartum psychosis)	100		6 months	ICD-8, ICD-10	Denmark	Danish registry / population study - women born in Denmark to Danish-born parents between January 1980 and December 1998 without previous psychiatric diagnoses who gave birth between 1995 and 2012	Obstetric complications	Registry	No association between any pregnancy/obstetrical complication and risk of postpartum psychosis including pre-eclampsia [IRR = 0.95 (95% CI: 0.46–1.97)], eclampsia [IRR = 0.00 (95% CI: 0.00–0.00)], gestational diabetes [IRR = 0.67 (95% CI: 0.17–2.74)], gestational hypertension [IRR = 0.99 (95% CI: 0.31–3.16)], hyperemesis gravidarum [IRR = 1.36 (95% CI: 0.43–4.27)], fetal stress [IRR = 1.00 (95% CI: 0.69–1.44)], postpartum hemorrhage [IRR = 0.96 (95% CI: 0.53–1.75)], Cesarean section [IRR = 1.21 (95% CI: 0.82–1.78)], preterm birth [IRR = 1.05 (95% CI: 0.65–1.69)], still birth [IRR = 0.00 (95% CI: 0.00–0.00)], previous abortion [IRR = 0.81 (95% CI: 0.56–1.18)].
Meltzer-Brody 2018 (36)	85,080 (31 postpartum psychosis)	100	6 months	ICD-8, ICD-10	Denmark	Danish registry / population study - all women born in Denmark on 1 January 1955 or later, who gave birth to a singleton, live-born child between 1 January 1995 and 30 June 2012.	Early adverse life events	Registry	Across all studied adversities, no increased risk of postpartum psychosis. Hazard ratios for family disruption [HR = 0.99 (95% CI: 0.41–2.37)], parental somatic illness [HR = 1.00 (95% CI: 0.35–2.86)], parental labor market exclusion [HR = 1.56 (95% CI: 0.52–4.63)], parental criminality [HR = 0.37 (95% CI: 0.05–2.85)], placement in out-of-home care [HR = 1.48 (95% CI: 0.31–7.12)], parental psychopathology excluding substance use disorder [HR = 0.91 (95% CI: 0.20–4.10)]. Too few exposed cases for parental death or parental substance use disorder to reliably estimate hazard ratios. Relative to experiencing 0 events, risk of postpartum psychosis was not significantly associated with exposure to 1 event [HR = 0.84 (95% CI: 0.35–2.05)], 2 events [HR = 1.49 (95% CI: 0.53–4.17)] or 3 or more events [HR = 0.72 (95% CI: 0.16–3.32)].
Nager 2008 (38)	1,133,368 (1,413 postpartum psychosis)		65	3 months	ICD-8, ICD-9	Sweden	All Swedish first-time mothers between 1 January 1975 and 31 December 2003, obtained by linking national population registers to the Medical Birth Register and the Hospital Discharge Register.	Obstetric complications	Registry	In the fully adjusted model, (adjusted for age, year of delivery and previous hospitalization for psychiatric disorder) only preterm birth [HR = 1.20 (95% CI: 1.01–1.44)] was significantly associated with postpartum psychosis. The following were not significant in the fully adjusted model: preeclampsia [HR = 1.13 (95% CI: 0.84–1.53)], obstetric trauma of the mother [HR = 1.04 (95% CI: 0.83–1.30)], postpartum hemorrhage [HR = 0.87 (95% CI: 0.67–1.13)], birth trauma in the neonate [HR = 0.97 (95% CI: 0.73–1.29)], respiratory disorder in the neonate [HR = 1.18 (95% CI: 0.99–1.40)], severe birth asphyxia [HR = 1.26 (95% CI: 0.90–1.77)], mild/moderate birth asphyxia [HR = 1.11 (95% CI: 0.93–1.32)], neonatal jaundice [HR = 0.94 (95% CI: 0.75–1.19)], post-term birth [HR = 0.80 (95% CI: 0.49–1.29)], multiple birth [HR = 0.84 (95% CI: 0.47–1.52)], Cesarean section [HR = 1.13 (95% CI: 0.99–1.30)], perinatal death [HR = 1.18 (95% CI: 0.75–1.86)], small-for-gestational-age infant [HR = 1.15 (95% CI: 0.92–1.43)]. In the partially adjusted model (adjusted for age and year of delivery), respiratory disorder in the neonate [HR = 1.27 (95% CI:1.07–1.51)], severe birth asphyxia [HR = 1.39 (95% CI: 1.00–1.95)], preterm birth [HR = 1.46 (95% CI: 1.22–1.74)], Cesarean section [HR = 1.32 (95% CI: 1.15–1.51)], perinatal death [HR = 1.59 (95% CI: 1.01–2.50)], and small-for-gestational-age infant [HR = 1.42 (95% CI: 1.17–1.77)] were all significantly associated with postpartum psychosis.
Vikstrom 2017 (44)	29,036 (106 postpartum psychosis)		99.6	1 year	ICD-10	Sweden	Individuals who received IVF between 1st January 1988 to 31st December 2012 and who were born from 1st January 1973 to 31st December 1991. Control group of primiparous women with spontaneous conception randomly selected from Swedish Medical Birth Register.	IVF	Registry	No difference in prevalence of postpartum psychosis between those undergoing IVF and those not (0.3% vs. 0.4%, Chi square test, *p* = 0.169), in multivariable analysis the odds of postpartum psychosis were no different in controls relative to IVF group [OR = 1.18 (95% CI: 0.59–2.37)].
Warselius 2019 (45)	4,174,311 (1,488 postpartum psychosis)		100	90 days	ICD-8, ICD-9, ICD-10	Denmark and Sweden	Registry study of live births in Denmark during 1978–2008 from Danish Medical Birth Register and births in Sweden during 1973–2006 from the Swedish Medical Birth Register	Death of close relative the year before or during pregnancy	Registry	No association between death of close relative and risk of first episode postpartum psychosis, adjusted [HR = 1.02 (95% CI 0.76–1.37)].

DSM, Diagnostic and Statistical Manual, International Classification of Disease; OR, Odds Ratio; CI, Confidence Interval; HR, Hazard Ratio; RR, Relative Risk; IRR, Incidence Rate Ratio; IVF, In Vitro Fertilization.

**Table 3:** Summary of cohort study examining subsequent relapse in women previously diagnosed with postpartum psychosis.

Study	Number of participants	Percentage first episode	Postpartum period	Diagnosis	Country	Setting	Adverse event	Measures	Findings
Terp 1999 (41)	600 (332 relapses, study reported an additional 9 cases of postpartum schizophrenia who were not analyzed in the results)	100	90 days	ICD-8	Denmark	Registry / population study, Danish Medical Birth Register - women admitted to a Danish psychiatric hospital between 1973 and 1993 with a psychotic episode less than 91 days after delivery	Obstetric complications	Registry	Preterm delivery was associated with reduced risk of readmission [RR = 0.6 (95% CI: 0.4–0.98)]. No significant differences for other stressors: Cesarean section [RR = 0.9 (95% CI: 0.6–1.3)], stillbirth [RR = 0.7 (95% CI: 0.4–1.5)], birth weight > 2,500 g versus <2,500 g [RR = 1.0 (95% CI: 0.6–1.6)], multiple births [RR = 1.0 (95% CI: 0.8–1.2)].

ICD, International Classification of Diseases; RR, Relative Risk; CI, Confidence Interval.

Across studies, a wide range of adverse life events were examined. These were broadly categorized as individual psychosocial stressors experienced by the mother (either in childhood or adulthood), life event checklists, complications of pregnancy, complications of birth, and complications in the neonate.

### 3.1. Individual psychosocial stressors experienced by the mother

Three individual adult psychosocial stressors were each examined by a single large population-based cohort study: previous abortion (37), *in vitro* fertilization (44), and death of a close relative in the year before or during pregnancy (45). None showed an association with postpartum psychosis. Meltzer-Brody et al. (37) examined previous abortion in a Danish population-based study of 392,458 women, and found no association with the onset of postpartum psychosis [RR = 0.81 (95% CI: 0.56–1.18)]. Vikstrom et al. (44) compared rates of postpartum psychosis among women who had become pregnant by *in vitro* fertilization with primiparous women experiencing spontaneous conception who were randomly selected from the Swedish Medical Birth Register. In the *in vitro* fertilization sample, 0.3% developed postpartum psychosis, compared with 0.4% of the spontaneous conception sample [OR = 1.18 (95% CI: 0.59–2.37)]. In a further registry study using data from Denmark and Sweden, Warselius et al. (45) investigated death of a close relative in the year before or during pregnancy but found no significant association with onset of postpartum psychosis [adjusted HR = 1.02 (95% CI: 0.76–1.37)].

Individual childhood adverse events were examined in one population-based cohort study of 85,080 women in Denmark, of whom 31 developed postpartum psychosis (36). None of the risk factors examined (family disruption, parental somatic illness, parental labour market exclusion, parental criminality, placement in out-of-home care, parental psychopathology) were significantly associated with onset of postpartum psychosis. Moreover, relative to experiencing no adverse events, postpartum psychosis was not associated with experiencing one, two, or more than two adverse events.

### 3.2. Life event checklists

Three studies (27, 32, 33) used life event checklists to measure adverse events, these measures included the List of Threatening Experiences (46), Life Events and Difficulties Schedule (47), and Interview for Recent Life Events (48).

Aas et al. (27) reported a positive association between life events and postpartum psychosis in the UK, though this was a small study with a case–control design. This study included women with postpartum psychosis, women at risk of postpartum psychosis (due to a previous diagnosis of bipolar disorder or schizoaffective disorder) who remained well, and healthy controls. Mean scores on the List of Threatening Experiences questionnaire were 3.5 (SD 3.4) in postpartum psychosis group, 2.2 (SD 3.2) in at risk group who did not develop postpartum psychosis, and 0.7 (SD 1.7) in healthy controls. There was a significant effect of group status on scores (*F* = 4.3, *p* = 0.019), with pairwise comparisons showing that mean life event scores were statistically different in the postpartum psychosis group and healthy controls only (*p* = 0.007).

In another small UK case–control study, Brockington et al. (32) found a negative association between life events assessed using the Life Events and Difficulties Schedule, where women with postpartum psychosis were less likely to have experienced severe events or major difficulties in the 38 weeks prior to interview (*χ*^2^ = 4.9, *p* = 0.026). A further UK case–control study conducted by Dowlatshahi and Paykel (33) used the Interview for Recent Life Events (examining events in the past 13 months) and found no statistically significant differences in the number of recent life events experienced by women with postpartum psychosis and healthy controls.

### 3.3. Complications of pregnancy

Pre-eclampsia was investigated by four studies, none of which showed a significant association with postpartum psychosis. These included two large registry studies in Denmark (37) and Sweden (38), in addition to a cohort study in the UK (34) and a case–control study in the USA (39).

Two case–control studies based in Denmark (43) and India (42) examined any maternal complication during pregnancy, neither of which observed an association with onset of postpartum psychosis. One case–control study in the USA (39) observed that maternal respiratory illnesses were significantly more common among women with postpartum psychosis than healthy controls (*p* < 0.05). All other complications of pregnancy were examined by single studies and did not show significant associations: antepartum hemorrhage (34), anemia in pregnancy (34), malposition *in utero* (34), hydramnios (34), renal/urinary infection (34), gestational diabetes (37), gestational hypertension (37), hyperemesis gravidarum (37), fetal stress (37), placenta praevia (39), and abruptio placentae (39).

### 3.4. Complications of birth

The most common complication of birth was Cesarean section, examined in six studies (two case–control and four cohort). Allwood et al. (30) found a lower incidence of Cesarean section among women with postpartum psychosis compared with healthy controls (neither incidence rates nor statistical tests reported); however, the authors interpreted this as a possible artifact due to the method of recruiting the controls (who were identified from follow-up clinic appointments). In a small (*n* = 119) case–control study in the Netherlands, Bergink and colleagues (31) similarly observed that Cesarean section was less common in women with postpartum psychosis (9.5%) compared with healthy controls (25%), which was statistically significant (*p* = 0.03). In contrast, Kendell et al. (34), who examined a cohort including all live births in Edinburgh between 1971 and 1977, reported that Cesarean section was associated with an increased risk of postpartum psychosis: occurring in 21.6% women who went on to develop postpartum psychosis and 8.4% of the control population (*p* < 0.01). Notably, the two largest studies (37, 38), both nationwide register studies with low risk of bias, observed no significant association between Cesarean section and risk of onset of postpartum psychosis. Non-significant associations between Cesarean section and postpartum psychosis onset were also reported by Nager et al. (38), in a model adjusting for age, year of delivery, and previous hospitalization for psychiatric disorder [adjusted HR = 1.13 (95% CI: 0.99–1.30)] and Meltzer-Brody et al. (37) [RR = 1.21 (95% CI: 0.82–1.78)]. Consistent with these findings, the only study examining relapse of postpartum psychosis (41), found no association with Cesarean section [RR = 0.9 (95% CI: 0.6–1.3)].

Pre-term birth was examined by three studies. Videbech et al. (43), using a case–control study design, found that preterm birth was more common in women with postpartum psychosis (27%) than healthy controls (9%), which remained statistically significant after adjusting for parity (OR = 3.6, *p* < 0.01). Similarly, a large population cohort study using Swedish registers (38) observed that pre-term birth was significantly associated with the onset of postpartum psychosis after adjusting for age, year of delivery, and previous hospitalization for psychiatric disorder [adjusted HR = 1.20 (95% CI: 1.01–1.44)]. However, a large Danish cohort study failed to replicate this finding (37), instead observing no association between preterm birth and postpartum psychosis onset [HR = 1.05 (95% CI: 0.65–1.69)]. In contrast, Terp et al. (41), reported a negative association between preterm birth and subsequent relapse in women who had been previously diagnosed with first onset postpartum psychosis [RR = 0.6 (95% CI: 0.4–0.98)].

Postpartum hemorrhage was examined by three cohort studies conducted in Denmark (37), Sweden (38), and the UK (34); none found an association with this risk factor and onset of postpartum psychosis. Length of labor was positively associated with postpartum psychosis in a small (n = 38) case–control study (40) (mean duration of labor 11.15 h in the puerperal psychosis group vs. 7.34 h in the control group, one sided t test *p* < 0.05), but showed no association in another, larger case control study (*n* = 150) (43). A large registry cohort study based in Sweden (38) and another case–control study in Denmark (43) examined post-term birth, with neither finding an association with postpartum psychosis onset. Two studies examined malpresentation of fetus (34, 39), with neither finding an association.

Most types of birth complication were examined by a single study. Those showing significant associations with onset of postpartum psychosis included longer mean length of hospital stay (7.4 days in cases vs. 3.5 days in controls, *p* < 0.001) in a study conducted in South Africa (30); total dystocia (occurring in 14% in postpartum psychosis vs. 5% of controls, *p* < 0.05) in a case–control study in the USA (39); and night-time delivery (occurring in 71% of women with puerperal psychosis vs. 41% of controls, p < 0.05) in one case–control study in Canada (40). When considering any maternal complication during perinatal period, a case–control study in India found this was significantly more common among women with postpartum psychosis than controls (38% vs. 22%, respectively, *p* = 0.01) (42). The following birth complications were found to show no association with onset of postpartum psychosis: vacuum extraction (31), forceps delivery (34), abnormality of bony pelvis (34), prolonged labor (34), laceration of perineum (34), sepsis (34), pyrexia of unknown origin (34), puerperal anemia (34), eclampsia (37), obstetric trauma of the mother (38), multiple birth (38), prolapsed cord (39), cephalopelvic disproportion (39), uterine dysfunction (39), Cesarean or instrumental delivery (42), any birth complications or surgical interventions (43).

### 3.5. Complications of neonate

Four studies examined perinatal death (including stillbirth). Paffenbarger et al. (39) reported significantly more fetal or neonatal deaths in women with postpartum psychosis compared to controls (7% vs. 3%, *p* = 0.02). In a large study comprising all hospital births in Florida state between 2005 and 2014, Lewkowitz et al. (35) observed that subsequent onset of postpartum psychosis was more common among women who experienced stillbirth (0.9%) compared with the livebirth group (0.4%). In this study, stillbirth was significantly associated with postpartum psychosis in a multivariable model adjusted for maternal age, race/ethnicity, insurance type, income quartile, mode of delivery, severe intrapartum maternal morbidity, and medical comorbidities. However, this was not found in other studies. Kendell et al. (34) failed to find an association between perinatal death and onset of postpartum psychosis in a UK cohort (2.8% of the postpartum psychosis group vs. 1.7% of the control group), while a large registry cohort study in Sweden (38) also failed to find an association in a model adjusted for age, year of delivery, and previous hospitalization [adjusted HR = 1.18 (95% CI: 0.75–1.86)].

There was a higher proportion of neonates requiring intensive care (40.7% in postpartum psychosis and 24.5% of healthy controls, *p* < 0.01) in a single case–control study in South Africa (30). Similarly, a single case–control study in India observed that complications in the neonate were more common among women with postpartum psychosis compared to controls (21% vs. 8%, respectively, *p* = 0.009) (42). Videbech et al. (43) reported that the mean birthweight was lower in the postpartum psychosis group relative to controls (3,204 g vs. 3,523 g, *p* < 0.05).

The following complications of the neonate that were examined by single studies were not found to be significantly associated with postpartum psychosis: birth trauma in the neonate (38), respiratory disorder in the neonate (38), severe birth asphyxia (38), mild/moderate birth asphyxia (38), neonatal jaundice (38), and small for gestational age (38).

### 3.6. Summary of evidence across all studies

**Figure 2:**
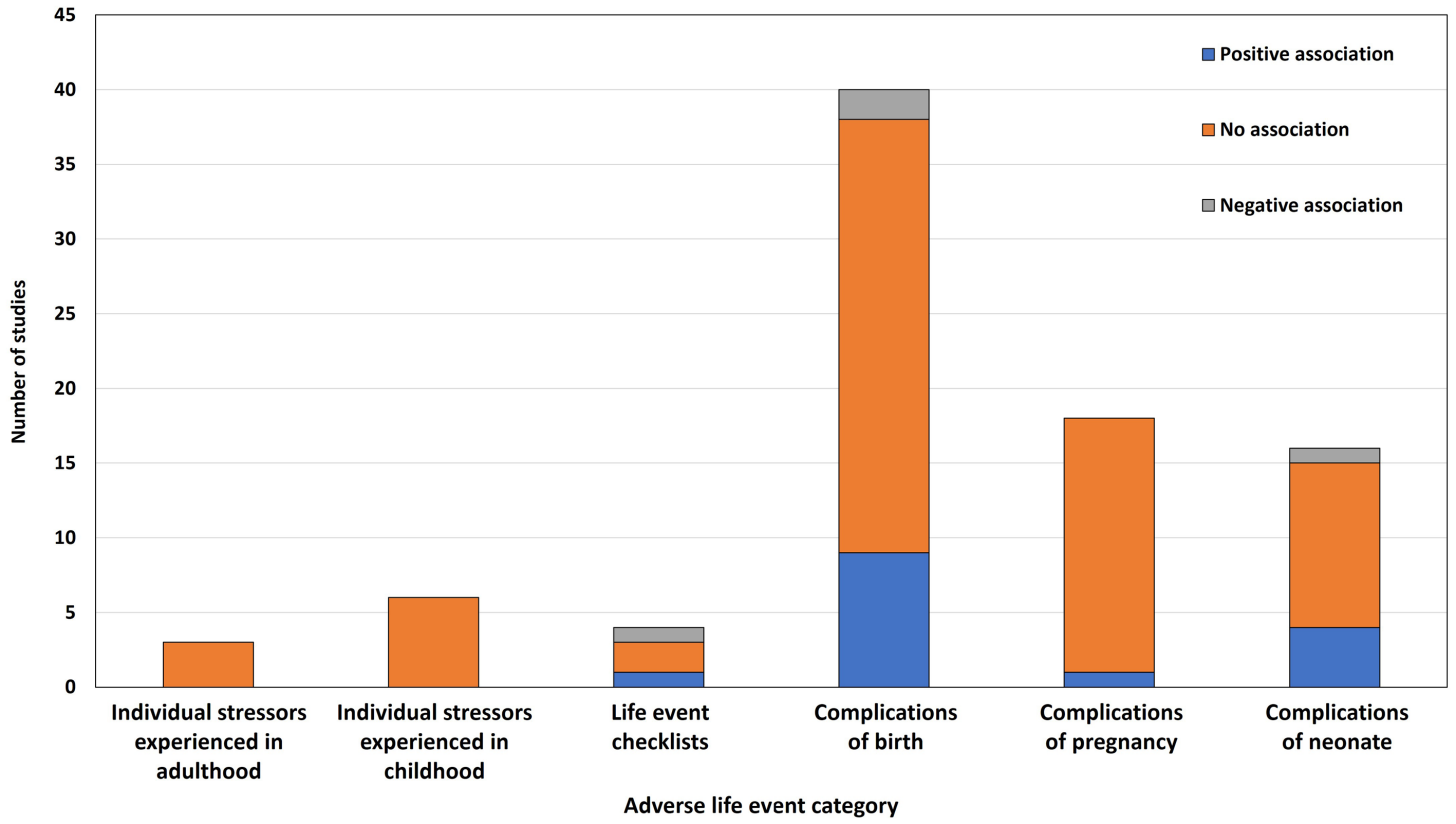
Frequency of reported associations across studies, grouped by type of adverse event.

Figure 2 summarizes the results of the reported associations across studies, grouped by category of adverse event. Overall, 63 different measures of adversity were examined and 87 associations between these measures and postpartum psychosis were tested. In terms of statistically significant associations with onset/relapse of postpartum psychosis, 15 (17%) were positive (i.e., the adverse event increased the risk of onset/relapse), 4 (5%) were negative (i.e., the adverse event decreased the risk of onset/relapse), and 68 (78%) were not statistically significant.

### 3.7. Risk of bias

Risk of bias ratings for case–control studies are shown in Table 4. Total scores for case–control studies ranged between 3 and 9, with a median of 7.0 (IQR = 4.0). Only two studies (27, 32) took adequate steps to ensure that controls did not have postpartum psychosis or any other mental disorder. No studies demonstrated that non-response rates were equivalent in cases and controls. Aas et al. (27) and Videbech et al. (43) scored highest with 9, of note, both studies had adequate definition of cases, unbiased selection of cases, selection of representative controls, unbiased selection of controls, adjusted/matched for confounders, and the same method of ascertaining exposure in cases and controls. Three studies (Allwood et al. (30), Bergink et al. (31) and Upadhyaya et al. (42)) obtained low scores (each scoring 3/12). None of these studies took steps to ensure selected controls were representative.

**Table 4:** Risk of bias in case–control studies.

Study	Case definition adequate? Max 1	Cases are representative? Max 1	Selection of cases unbiased? Max 1	Controls do not have the outcome of interest? Max 1	Controls representative? Max 1	Control group unbiased? Max 1	Cases and controls are matched and/or adjustment for confounders? Max 2	Exposure ascertainment robust, uses reliable/valid measure? Max 2	Same method of ascertaining exposure in cases and controls? Max 1	Non-response rates same in case and controls? Max 1	Total score. Max 12
Aas 2020 (27)	1	0	1	1	1	1	2	1	1	0	9
Allwood 2000 (30)	0	0	0	0	0	0	2	1	0	0	3
Bergink 2013 (31)	1	0	1	0	0	1	0	0	0	0	3
Brockington 1990 (32)	1	0	1	1	1	1	0	1	1	0	7
Dowlatshahi 1990 (33)	0	1	1	0	0	1	2	1	1	0	7
Paffenbarger 1961 (39)	0	1	1	0	1	1	1	0	1	0	6
Sharma 2004 (40)	0	1	1	0	0	1	2	1	1	0	7
Upadhyaya 2014 (42)	0	1	1	0	0	0	0	0	1	0	3
Videbech 1995 (43)	1	1	1	0	1	1	2	1	1	0	9

Max: maximum score, studies could score a maximum of 1 or 2 for individual questions and a maximum of 12 overall.

Table 5 shows risk of bias scores for cohort studies. Overall, scores were higher for cohort studies (range: 6–11, median of 8.5, IQR = 4.0) than for the case–control studies (indicating lower risk of bias). All studies were determined to have used a representative cohort and ensured the non-exposed and exposed groups were taken from the same population. Moreover, all employed robust methods to ascertain exposure, had limited risk of observer bias for the outcome, and had a sufficiently long time-period for the outcome to occur. However, none of the studies adequately addressed loss to follow-up in that they failed to report the proportion who were not followed up and whether attrition was associated with exposure status. That said, several studies (36–38, 45) noted that individuals were followed until death or emigration and used appropriate statistical models to account for censoring. The highest scoring study (11/12) was Meltzer-Brody et al. (37), this study examined obstetric complications using a population-based cohort design and was notable as it was one of only two studies that adequately adjusted for potential confounders. The lowest scoring study was Kendell et al. (34), which was also the earliest cohort study and had several limitations including failure to ensure the outcome was not present at the start of the study, failure to adjust for potential confounders, and failure to report loss to follow-up.

**Table 5:** Risk of bias in cohort studies.

Author	Exposed cohort is representative? Max 1	Non-exposed group selected from same population? Max 1	Ascertainment exposure of robust? Max 1	Outcome not present at the start? Max 1	Exposed and unexposed are matched or adjustment for confounding? Max 2	Assessment of outcome has limited potential for information (observer) bias Max 1	Assessment of outcome is robust? Max 2	Follow-up sufficiently long for outcomes to occur? Max 1	Loss to follow-up rate is reported, low (<30%), and not associated with exposure status? Max 2	Total score. Max 12
Kendell 1981 (34)	1	1	1	0	0	1	1	1	0	6
Lewkowitz 2019 (35)	1	1	1	1	2	1	2	1	0	10
Meltzer-Brody 2017 (37)	1	1	1	1	2	1	2	1	1	11
Meltzer-Brody 2018 (36)	1	1	1	1	1	1	1	1	1	9
Nager 2008 (38)	1	1	1	0	1	1	1	1	1	8
Terp 1999 (41)	1	1	1	1	1	1	1	1	0	8
Vikstrom 2017 (44)	1	1	1	0	2	1	1	1	0	8
Warselius 2019 (45)	1	1	1	1	2	1	1	1	1	10

Max: maximum score, studies could score a maximum of 1 or 2 for individual questions and a maximum of 12 overall.

## 4. Discussion

To our knowledge, this is the first systematic review to examine the association between adverse life events and postpartum psychosis. Our review identified 17 studies examining a wide range of adverse life events experienced in childhood and adulthood, as well as those specific to the perinatal period. While these studies failed to convincingly show that adverse life events (experienced at any stage of life) contributed to the onset or relapse of postpartum psychosis, our review identified several major limitations with the current evidence base which prevents us from drawing meaningful conclusions.

One important limitation was that most risk factors (52/63) were examined in a single study only; this was particularly the case for adverse events occurring outside of perinatal period. Although some studies used population-based registry designs (36, 37, 44, 45), the number of participants who developed postpartum psychosis was still relatively low. Furthermore, these record linkage studies defined the postpartum period as ranging from 3 months to 1 year, which is considerably longer than current DSM or ICD definitions of postpartum: 4 and 6 weeks, respectively.

Replications of the results are required before concluding that adverse life events in childhood or adulthood show no association with postpartum psychosis. Indeed, one study that did not meet our inclusion criteria suggested an association is possible: Hazelgrove et al. (28), in a small case–control study, reported that severe childhood maltreatment was associated with postpartum relapse in an at-risk sample. Another study (49) that did not meet inclusion criteria suggested that there was no difference in the rate of childhood adverse events between patients with bipolar disorder who did or did not experience postpartum relapse.

There may also be differences in perceived stress in women vulnerable to postpartum psychosis. Aas et al. (27) observed that women with postpartum psychosis reported higher levels of perceived stress compared with controls, while Allwood et al. (30) also reported increased levels of perceived stress in their postpartum psychosis group. This suggests that while rates of adverse events may be similar among women experiencing postpartum psychosis and healthy controls, women with postpartum psychosis may perceive these events as more stressful and/or uncontrollable.

Perinatal complications are not conventionally considered as adverse life events in psychosis research. These were included to be as broad as possible in our search. Our findings are similar to a previous systematic review of peripartum complications in relation to postpartum psychosis (23), which showed a lack of replication for any single risk factor. Notably this previous systematic review had limited information on included studies, did not formally assess study quality, and included study designs with high risk of bias, such as case reports.

A lack of association between adverse life events and postpartum psychosis has been noted in previous narrative reviews (4, 19, 20), but this is the first systematic review to comprehensively search across a broad range of risk factors. Given the evidence from research published to date, identifying individuals at-risk for postpartum psychosis should focus on well-established risk factors of large effect, such as personal or family history of postpartum psychosis, or personal history of bipolar disorder. The lack of association with adverse life events contrasts with postpartum depression, where adverse life events are among the strongest predictors (21, 50). It also differs from psychotic disorders that onset outside the postpartum period, which have been associated with increased exposure to adverse life events (16). As noted previously, postpartum psychosis is conceptualized as part of the bipolar spectrum, thus, it is notable that childhood adversity is associated with bipolar disorder (51).

Given the absence of evidence supporting a consistent association between adverse life events and postpartum psychosis, other etiological mechanisms beyond the role of adverse events should also be examined in future research. One important risk factor to consider is sleep disturbance, which is a recognized feature of bipolar disorder and schizophrenia (52). Women with bipolar disorder who experience episodes triggered by sleep loss are twice as likely to have experienced an episode of postpartum psychosis compared with women without this trigger (53). Thus, sleep loss associated with childbirth could be a causative factor for some women with this vulnerability.

Female sex hormones that show major fluctuations during the postpartum period might also play a contributing role. Indeed estrogen, which (along with progesterone) increases during pregnancy and then precipitously falls after birth (19), has been hypothesized to be protective against psychosis (54) with withdrawal states associated with psychotic episodes (55). Despite the plausibility of this potential mechanism, few studies have directly measured or manipulated hormones in postpartum psychosis. Of note, a single open label trial of prophylactic estradiol for the prevention of postpartum psychosis in women with a bipolar disorder yielded negative findings (56).

There is some evidence to suggest that genetic predisposition contributes to postpartum psychosis. Women with bipolar disorder who experienced postpartum psychosis were more likely to report a history of affective disorder in first-degree relatives than those with bipolar disorder in the absence of postpartum episodes (57). Moreover, one study suggests that postpartum psychosis shares polygenetic liability with both bipolar disorder and schizophrenia but not major depression (5).

Dysfunction of the immune system has been postulated as a potential mechanism of postpartum psychosis (58). Pregnancy and postpartum are associated with immunomodulation that allows tolerance of the fetus while also providing protection from pathogens. Key inflammatory markers that have been associated with postpartum psychosis are interleukin-8, monocyte chemoattractant protein-1 and c-reactive protein (58). These markers are also associated with psychotic disorders outside the postpartum period (59).

### 4.1. Limitations

There are some limitations that should be taken into consideration. We chose to focus on first-episode postpartum psychosis given that a prior postpartum psychosis, bipolar disorder, and schizoaffective disorder are established risk factors, with the potential to confound the relationship. However, in some studies, the samples included patients who were not first episode cases, and in three (33, 34, 40) the number who were experiencing a first episode was unclear. We chose to include a broad range of adverse events, which incorporated both psychosocial stressors and biological factors, such as IVF. We therefore included adverse events that may not be considered as relevant in most studies of psychosis outside the postpartum period.

The studies we examined were predominately from European or North American populations. Only one was from Africa and one from Asia, and there were no studies from South America. The large record-linkage studies which showed a lack of association for a variety of adverse life events in adequately powered samples are from only two countries, Sweden and Denmark. This limits the generalizability of our conclusions beyond Western populations.

We identified only one study that examined the effect of adverse events on relapse following a diagnosis of postpartum psychosis (41), all others examined onset. The definition of ‘postpartum’ varied between studies, with some considering any psychotic episode within 1 year of childbirth. This is at odds with DSM and ICD classifications of the postpartum period, which are defined as 4 and 6 weeks, respectively. Due to the heterogeneity in study design, risk factors and outcomes, we were unable to perform a meta-analysis to pool results across studies.

### 4.2. Conclusion

In this systematic review, which included studies examining a wide range of risk factors, we were unable to identify robust evidence of an association between adverse life events and onset of postpartum psychosis. Notably, of the 63 potential risk factors studied, only 11 (17%) were examined by more than one study. Further replication, using consistent definitions of postpartum psychosis and risk factors, is required to establish whether there is truly an absence of association.

Studies examining relapse following a diagnosis of postpartum psychosis were also lacking (indeed only one study was identified). Given that more than half of women with first-onset postpartum psychosis will experience further episodes outside the postpartum period (11), and are also at risk of suicide (60), understanding factors that contribute to illness relapse is crucial to improving outcomes for women with this disorder.

Future studies should address the major limitations that we have identified in this review, notably, using a consistent definition of postpartum that corresponds with established diagnostic systems (DSM and ICD). Given the lack of studies examining the contribution of childhood adversity (a well-established risk factor for psychotic disorders generally), we strongly recommend that future studies in this field investigate these measures.

## Author contributions

AEC designed and supervised the study. ER conducted the search, screened studies, extracted data, conducted bias ratings, and wrote parts of the manuscript. TJR extracted data, conducted bias ratings, and drafted the manuscript. VCSB screened studies and extracted data. AEC, PM, and PD critically revised the manuscript. All authors contributed to the article and approved the submitted version.

## Funding

TJR is supported by an MRC Clinical Research Training Fellowship, MR/W015943/1. AEC is supported by a NARSAD Young Investigator Grant awarded by the Brain & Behavior awarded by the Brain & Behavior Research Foundation, 28336.

## Conflict of interest

The authors declare that the research was conducted in the absence of any commercial or financial relationships that could be construed as a potential conflict of interest.

## Publisher’s note

All claims expressed in this article are solely those of the authors and do not necessarily represent those of their affiliated organizations, or those of the publisher, the editors and the reviewers. Any product that may be evaluated in this article, or claim that may be made by its manufacturer, is not guaranteed or endorsed by the publisher.

## References

[1] Jones I Chandra PS Dazzan P Howard LM. Bipolar disorder, affective psychosis, and schizophrenia in pregnancy and the post-partum period. Lancet. (2014) 384:1789–99. doi: 10.1016/S0140-6736(14)61278-2, PMID: 25455249

[2] Vander Kruik R Barreix M Chou D Allen T Say L Cohen LS. The global prevalence of postpartum psychosis: a systematic review. BMC Psychiatry. (2017) 17:1–9. doi: 10.1186/s12888-017-1427-7 28754094 PMC5534064

[3] Munk-Olsen T Jones I Laursen TM. Birth order and postpartum psychiatric disorders. Bipolar Disord. (2014) 16:300–7. doi: 10.1111/bdi.12145 24636279

[4] Perry A Gordon-Smith K Jones L Jones I. Phenomenology epidemiology and aetiology of postpartum psychosis: a review. Brain Sci. (2021) 11:1–14. doi: 10.3390/brainsci11010047 PMC7824357 33406713

[5] Di Florio A Mei Kay Yang J Crawford K Bergink V Leonenko G Pardiñas AF. Post-partum psychosis and its association with bipolar disorder in the UK: a case-control study using polygenic risk scores. The lancet. Psychiatry. (2021) 8:1045–52. doi: 10.1016/S2215-0366(21)00253-4, PMID: 34715029

[6] Kamperman AM Veldman-Hoek MJ Wesseloo R Robertson Blackmore E Bergink V. Phenotypical characteristics of postpartum psychosis: a clinical cohort study. Bipolar Disord. (2017) 19:450–7. doi: 10.1111/bdi.12523, PMID: 28699248

[7] Munk-Olsen T Laursen TM Mendelson T Pedersen CB Mors O Mortensen PB. Risks and predictors of readmission for a mental disorder during the postpartum period. Arch Gen Psychiatry. (2009) 66:189–95. doi: 10.1001/archgenpsychiatry.2008.528, PMID: 19188541

[8] Appleby L Mortensen PB Faragher EB. Suicide and other causes of mortality after post-partum psychiatric admission. Br J Psychiatry. (1998) 173:209–11. doi: 10.1192/bjp.173.3.209, PMID: 9926095

[9] Chin K Wendt A Bennett IM Bhat A. Suicide and maternal mortality. Curr Psychiatry Rep. (2022) 24:239–75. doi: 10.1007/s11920-022-01334-3, PMID: 35366195 PMC8976222

[10] Blackmore ER Rubinow DR O'Connor TG Liu X Tang W Craddock N. Reproductive outcomes and risk of subsequent illness in women diagnosed with postpartum psychosis. Bipolar Disord. (2013) 15:394–404. doi: 10.1111/bdi.12071, PMID: 23651079 PMC3740048

[11] Gilden J Kamperman AM Munk-Olsen T Hoogendijk WJG Kushner SA Bergink V. Long-term outcomes of postpartum psychosis: a systematic review and meta-analysis. J Clin Psychiatry. (2020):81. doi: 10.4088/JCP.19r12906 32160423

[12] Ripke S Neale BM Corvin A Walters JTR Farh KH Holmans PA. Biological insights from 108 schizophrenia-associated genetic loci. Nature. (2014) 511:421–7. doi: 10.1038/nature13595 25056061 PMC4112379

[13] Murray RM Bhavsar V Tripoli G Howes O. 30 years on: how the neurodevelopmental hypothesis of schizophrenia morphed into the developmental risk factor model of psychosis. Schizophr Bull. (2017) 43:1190–6. doi: 10.1093/schbul/sbx121, PMID: 28981842 PMC5737804

[14] Stilo SA Murray RM. Non-genetic factors in schizophrenia. Curr Psychiatry Rep. (2019) 21:100. doi: 10.1007/s11920-019-1091-3, PMID: 31522306 PMC6745031

[15] Morgan C Gayer-Anderson C. Childhood adversities and psychosis: evidence, challenges, implications. World Psychiatry. (2016) 15:93–102. doi: 10.1002/wps.20330, PMID: 27265690 PMC4911761

[16] Beards S Gayer-Anderson C Borges S Dewey ME Fisher HL Morgan C. Life events and psychosis: a review and meta-analysis. Schizophr Bull. (2013) 39:740–7. doi: 10.1093/schbul/sbt065, PMID: 23671196 PMC3686461

[17] Gausia K Ryder D Ali M Fisher C Moran A Koblinsky M. Obstetric complications and psychological well-being: experiences of Bangladeshi women during pregnancy and childbirth. J Health Popul Nutr. (2012) 30:172–80. doi: 10.3329/jhpn.v30i2.11310, PMID: 22838159 PMC3397328

[18] Martland N Martland R Cullen AE Bhattacharyya S. Are adult stressful life events associated with psychotic relapse? A systematic review of 23 studies. Psychol Med. (2020) 50:2302–16. doi: 10.1017/S0033291720003554, PMID: 33054892

[19] Bergink V Rasgon N Wisner KL. Postpartum psychosis: madness, mania, and melancholia in motherhood. Am J Psychiatr. (2016) 173:1179–88. doi: 10.1176/appi.ajp.2016.16040454, PMID: 27609245

[20] Davies W. Understanding the pathophysiology of postpartum psychosis: challenges and new approaches. World J Biol Psychiatry. (2017) 7:77–88. doi: 10.5498/wjp.v7.i2.77, PMID: 28713685 PMC5491479

[21] Guintivano J Manuck T Meltzer-Brody S. Predictors of postpartum depression: a comprehensive review of the last decade of evidence. Clin Obstet Gynecol. (2018) 61:591–603. doi: 10.1097/GRF.0000000000000368, PMID: 29596076 PMC6059965

[22] Blom EA Jansen PW Verhulst FC Hofman A Raat H Jaddoe VWV. Perinatal complications increase the risk of postpartum depression. The generation R study. BJOG. (2010) 117:1390–8. doi: 10.1111/j.1471-0528.2010.02660.x, PMID: 20682022

[23] Nguyen K Mukona LT Nalbandyan L Yar N St. Fleur G Mukona L. Peripartum complications as risk factors for postpartum psychosis: a systemic review. Cureus. (2022) 14:1–7. doi: 10.7759/cureus.29224 PMC9495292 36159350

[24] Page MJ McKenzie JE Bossuyt PM Boutron I Hoffmann TC Mulrow CD. The PRISMA 2020 statement: an updated guideline for reporting systematic reviews. BMJ. (2020) 2021:372. doi: 10.1136/bmj.n71 PMC8005924 33782057

[25] American Psychiatric Association. Diagnostic and statistical manual of mental disorders. 5th ed. Arlington, VA: American Psychiatric Association (2013).

[26] World Health Organization. International statistical classification of diseases and related health problems. 11th ed. Geneva: WHO (2019).

[27] Aas M Vecchio C Pauls A Mehta M Williams S Hazelgrove K. Biological stress response in women at risk of postpartum psychosis: the role of life events and inflammation. Psychoneuroendocrinology. (2020) 113:104558. doi: 10.1016/j.psyneuen.2019.104558, PMID: 31923613

[28] Hazelgrove K Biaggi A Waites F Fuste M Osborne S Conroy S. Risk factors for postpartum relapse in women at risk of postpartum psychosis: the role of psychosocial stress and the biological stress system. Psychoneuroendocrinology. (2021) 128:105218. doi: 10.1016/j.psyneuen.2021.105218, PMID: 33892376

[29] Wells G Shea B O'Connell D Peterson J Welch V Losos M. (2011). The Newcastle-Ottawa scale (NOS) for assessing the quality of nonrandomised studies in meta-analyses. Available at: https://www.ohri.ca/programs/clinical_epidemiology/oxford.asp (Accessed March 23, 2023).

[30] Allwood CW Berk M Bodemer W. An investigation into puerperal psychoses in black women admitted to Baragwanath hospital. S Afr Med J. (2000) 90:518–20. 10901827

[31] Bergink V Burgerhout KM Weigelt K Pop VJ De Wit H Drexhage RC. Immune system dysregulation in first-onset postpartum psychosis. Biol Psychiatry. (2013) 73:1000–7. doi: 10.1016/j.biopsych.2012.11.006, PMID: 23270599

[32] Brockington IF Martin C Brown GW Goldberg D Margison F. Stress and puerperal psychosis. Br J Psychiatry. (1990) 157:331–4. doi: 10.1192/bjp.157.3.331 2245259

[33] Dowlatshahi D Paykel ES. Life events and social stress in puerperal psychoses: absence of effect. Psychol Med. (1990) 20:655–62. doi: 10.1017/S0033291700017177, PMID: 2236375

[34] Kendell RE Rennie D Clarke JA Dean C. The social and obstetric correlates of psychiatric admission in the puerperium. Psychol Med. (1981) 11:341–50. doi: 10.1017/S0033291700052156, PMID: 7267875

[35] Lewkowitz AK Rosenbloom JI Keller M López JD Macones GA Olsen MA. Association between stillbirth ≥23 weeks gestation and acute psychiatric illness within 1 year of delivery. Am J Obstet Gynecol. (2019) 221:491.e1-.e22–491.e22. doi: 10.1016/j.ajog.2019.06.027, PMID: 31226297 PMC6829063

[36] Meltzer-Brody S Larsen JT Petersen L Guintivano J Florio AD Miller WC. Adverse life events increase risk for postpartum psychiatric episodes: a population-based epidemiologic study. Depress Anxiety. (2018) 35:160–7. doi: 10.1002/da.22697, PMID: 29172228 PMC6867605

[37] Meltzer-Brody S Maegbaek ML Medland SE Miller WC Sullivan P Munk-Olsen T. Obstetrical pregnancy and socio-economic predictors for new-onset severe postpartum psychiatric disorders in primiparous women. Psychol Med. (2017) 47:1427–41. doi: 10.1017/S0033291716003020, PMID: 28112056 PMC5429203

[38] Nager A Sundquist K Ramírez-León V Johansson LM. Obstetric complications and postpartum psychosis: a follow-up study of 1.1 million first-time mothers between 1975 and 2003 in Sweden. Acta Psychiatr Scand. (2008) 117:12–9. doi: 10.1111/j.1600-0447.2007.01096.x 17941968

[39] Paffenbarger RS Jr Steinmetz CH Pooler BG Hyde RT. The picture puzzle of the postpartum psychoses. J Chronic Dis. (1961) 13:161–73. doi: 10.1016/0021-9681(61)90149-7, PMID: 13732004

[40] Sharma V Smith A Khan M. The relationship between duration of labour, time of delivery, and puerperal psychosis. J Affect Disord. (2004) 83:215–20. doi: 10.1016/j.jad.2004.04.014, PMID: 15555716

[41] Terp IM Engholm G Møller H Mortensen PB. A follow-up study of postpartum psychoses: prognosis and risk factors for readmission. Acta Psychiatr Scand. (1999) 100:40–6. doi: 10.1111/j.1600-0447.1999.tb10912.x, PMID: 10442438

[42] Upadhyaya SK Sharma A Raval CM. Postpartum psychosis: risk factors identification. N Am J Med Sci. (2014) 6:274–7. doi: 10.4103/1947-2714.134373, PMID: 25006563 PMC4083529

[43] Videbech P Gouliaev G. First admission with puerperal psychosis: 7–14 years of follow-up. Acta Psychiatr Scand. (1995) 91:167–73. doi: 10.1111/j.1600-0447.1995.tb09761.x, PMID: 7625190

[44] Vikström J Josefsson A Hammar M Bladh M Sydsjö G. Risk of postpartum psychosis after IVF treatment: a nationwide case-control study. Hum Reprod. (2017) 32:139–46. doi: 10.1093/humrep/dew302 27927846

[45] Warselius P Cnattingius S Li J Wei D Valdimarsdottir UA Kosidou K. Maternal bereavement shortly before or during pregnancy and risk of postpartum psychotic illness: a population-based study from Denmark and Sweden. Clin Epidemiol. (2019) 11:285–98. doi: 10.2147/CLEP.S195741, PMID: 31118817 PMC6500870

[46] Brugha T Bebbington P Tennant C Hurry J. The list of threatening experiences: a subset of 12 life event categories with considerable long-term contextual threat. Psychol Med. (1985) 15:189–94. doi: 10.1017/S003329170002105X, PMID: 3991833

[47] Brown GW Birley JL. Crises and life changes and the onset of schizophrenia. J Health Soc Behav. (1968) 9:203–14. doi: 10.2307/2948405, PMID: 5676853

[48] Paykel ES. Methodological aspects of life events research. J Psychosom Res. (1983) 27:341–52. doi: 10.1016/0022-3999(83)90065-X 6668560

[49] Perry A Gordon-Smith K Di Florio A Forty L Craddock N Jones L. Adverse childhood life events and postpartum psychosis in bipolar disorder. J Affect Disord. (2016) 205:69–72. doi: 10.1016/j.jad.2016.06.061, PMID: 27420133

[50] Biaggi A Conroy S Pawlby S Pariante CM. Identifying the women at risk of antenatal anxiety and depression: a systematic review. J Affect Disord. (2016) 191:62–77. doi: 10.1016/j.jad.2015.11.014, PMID: 26650969 PMC4879174

[51] Palmier-Claus JE Berry K Bucci S Mansell W Varese F. Relationship between childhood adversity and bipolar affective disorder: systematic review and meta-analysis. Br J Psychiatry. (2016) 209:454–9. doi: 10.1192/bjp.bp.115.179655, PMID: 27758835

[52] Meyer N Faulkner SM McCutcheon RA Pillinger T Dijk DJ MacCabe JH. Sleep and circadian rhythm disturbance in remitted schizophrenia and bipolar disorder: a systematic review and meta-analysis. Schizophr Bull. (2020) 46:1126–43. doi: 10.1093/schbul/sbaa024, PMID: 32154882 PMC7505194

[53] Lewis KJS Di Florio A Forty L Gordon-Smith K Perry A Craddock N. Mania triggered by sleep loss and risk of postpartum psychosis in women with bipolar disorder. J Affect Disord. (2018) 225:624–9. doi: 10.1016/j.jad.2017.08.054, PMID: 28889048

[54] Riecher-Rössler A. Oestrogens, prolactin, hypothalamic-pituitary-gonadal axis, and schizophrenic psychoses. Lancet Psychiatry. (2017) 4:63–72. doi: 10.1016/S2215-0366(16)30379-0, PMID: 27856396

[55] Mahé V Dumaine A. Oestrogen withdrawal associated psychoses. Acta Psychiatr Scand. (2001) 104:323–31. doi: 10.1111/j.1600-0447.2001.00288.x, PMID: 11722312

[56] Kumar C McIvor R Davies T Brown N Papadopoulos A Wieck A. Estrogen administration does not reduce the rate of recurrence of affective psychosis after childbirth. J Clin Psychiatry. (2003) 64:112–8. doi: 10.4088/JCP.v64n0202, PMID: 12633118

[57] Jones I Craddock N. Do puerperal psychotic episodes identify a more familial subtype of bipolar disorder? Results of a family history study. Psychiatr Genet. (2002) 12:177–80. doi: 10.1097/00041444-200209000-00011, PMID: 12218664

[58] Hazelgrove K. The role of the immune system in postpartum psychosis. Brain, Behav Immun Health. (2021) 18:100359. doi: 10.1016/j.bbih.2021.100359, PMID: 34704078 PMC8521124

[59] Pillinger T Osimo EF Brugger S Mondelli V McCutcheon RA Howes OD. A meta-analysis of immune parameters, variability, and assessment of modal distribution in psychosis and test of the immune subgroup hypothesis. Schizophr Bull. (2019) 45:1120–33. doi: 10.1093/schbul/sby160, PMID: 30407606 PMC6737479

[60] Brockington I. Suicide and filicide in postpartum psychosis. Arch Womens Ment Health. (2017) 20:63–9. doi: 10.1007/s00737-016-0675-8, PMID: 27778148 PMC5237439

